# Understanding Physical Activity Intentions in Physical Education Context: A Multi-Level Analysis from the Self-Determination Theory

**DOI:** 10.3390/ijerph17030799

**Published:** 2020-01-28

**Authors:** David Sánchez-Oliva, Athanasios Mouratidis, Francisco M. Leo, José L. Chamorro, Juan J. Pulido, Tomás García-Calvo

**Affiliations:** 1 Faculty of Sport Science, University of Extremadura, 10003 Cáceres, Spain; davidsanchez@unex.es (D.S.-O.); jjpulido@unex.es (J.J.P.); tgarciac@unex.es (T.G.-C.); 2 Department of Psychology, Bilkent University, Ankara 06800, Turkey; thanasis.mouratidis@gmail.com; 3 Faculty of Teaching Education, University of Extremadura, 10003 Cáceres, Spain; 4 Faculty of Sport Sciences, Universidad Europea de Madrid, 28670 Madrid, Spain; josemaria.lopez2@universidadeuropea.es

**Keywords:** need support, need satisfaction, motivation, physical activity promotion

## Abstract

Using self-determination theory as a framework, we aimed to study the relationships between perceived need support and need satisfaction with self-determined motivation and extracurricular physical activity intentions in the physical education (PE) classroom, including sex and out-of-school sport participation as moderators. Additionally, we aimed to test whether a need-supportive classroom environment in PE moderates these associations. Participants were 1259 students (556 males) aged between 12 to 16 years (Mage = 13.46 years; SD = 0.74) from 77 PE classes. At the student level we found (a) need satisfaction to predict positively autonomous motivation and negatively amotivation, and (b) autonomous motivation to predict positively and amotivation to predict negatively intentions to undertake extracurricular physical activities. At the classroom level, in need-supportive classes males benefit more than females in terms of increased autonomous motivation while females benefit more than males in terms of decreased amotivation. Finally, class-level perceived need support moderated (i.e., attenuated) the negative association between need satisfaction and amotivation and between amotivation and intentions. These results suggest a buffering role that a need-supportive classroom environment may have on students’ motivation and behavior.

## 1. Introduction

Physical inactivity has been growing to alarming rates in developed countries with 81% of the adolescents failing to meet the minimum amounts of physical activity (PA) recommended by the World Health Organization [1]. To address this problem, experts have pointed out Physical Education (PE) classes as a suitable context for the promotion of PA in adolescence [2]. In particular, the learning environment of the PE classroom, the degree to which the students satisfy their needs, their quality of motivation, and their attitudes toward PE have been highlighted as important predictors of PA intentions [3,4]. It remains unknown however whether a positive and caring learning environment relates to quality of motivation and extracurricular PA intentions in a similar way among both males and females and among students who already differ in PA levels. This is an interesting research question because we are unaware whether some students may benefit more (or less) from a need-supportive PE environment (i.e., that allocates resources to promote basic psychological needs satisfaction). 

### 1.1. Self-Determination Theory

To examine which factors related to PA intentions, numerous studies have relied on self-determination theory (SDT) [5]. According to SDT, motivation lies on a continuum that ranges from higher to lower levels of self-determination, which refers to the degree to which behaviors are volitionally undertaken. The highest degree of self-determination is called intrinsic motivation, and manifests when an adolescent performs an activity out of pleasure, that is, as an end in itself. A lesser form of self-determined motivation is identified regulation, which occurs when a person ascribes social and personal importance in the activity he or she performs. Next, is introjected regulation, which characterizes someone who carries out an activity to avoid feelings of guilt (that would appear if he or she did not do so), or to accumulate feelings of pride. An even less self-determined type is external regulation, which defines activities that people performed to get a reward or to avoid punishments. Intrinsic motivation and identified regulation represent autonomous motivation—that form of motivation where the person volitionally undertakes an activity. In contrast, introjected and external regulation represent controlled motivation—that form of motivation where one acts because of some internal or external psychological pressures. Finally, the lowest level of self-determination is amotivation, defined by the absence of intentions to undertake a behavior either because of helplessness or lack of interest; when amotivated, a subject does not know exactly why he or she keeps practicing. Previous studies in PE have shown autonomous motivation to relate to positive outcomes, such as effort, vitality, and intentions to undertake extracurricular PA, whereas controlled motivation and amotivation have been related to negative consequences, such as boredom or unhappiness [6].

SDT argues that autonomous motivation is promoted when the basic psychological needs for autonomy, competence, and relatedness are satisfied [5]. The need for autonomy refers to feelings of volition and the sense that one has personal control over one’s own behaviors; the need for competence represents a sense of being successful when dealing with a task; the need for relatedness is defined by feelings of connection and belongingness within the social context. Several studies conducted in the PE context have shown that who satisfied their needs report more autonomous and less controlled motivation or amotivation [6].

We ask, what is it that makes students well satisfied in PE which in turn makes them more autonomously motivated? The SDT claims that social context plays an important role in the promotion of need satisfaction [5]. In this regard a PE teacher can satisfy their students’ need for autonomy, by using specific strategies such as taking the students’ perspective, providing choices to them, and transferring to them some responsibility in decision-making [7]. Likewise, to satisfy their students’ need for competence, PE teachers should aim at optimizing students’ perception of skills through activities that are adjusted to their level; PE teachers should also provide sufficient time to their students to achieve the proposed goals, and deliver positive feedback to them by acknowledging their efforts and progress [7]. Finally, to satisfy their students’ need for relatedness, PE teachers should create learning environments that promote feelings of inclusion, integration, trust, and respect among their students and between themselves and their students [8]. Previous studies have found that students who perceive the learning environment of the PE class as need-supportive show greater need satisfaction and in turn more autonomous motivation, less amotivation, and more intentions to increase their PA levels by undertaking for instance some out-of-school sport activities [6].

In our study, we investigate whether the perceived learning environment and basic need satisfaction relate to the quality of motivation and extracurricular PA intentions in a similar way among males and females and among students who already differ in their PA levels. Investigating whether these relations are similar across all students or not would help us design more effective intervention programs in the PE class that will be especially designed for males (or females) or for those who are less active in PA.

### 1.2. The Role of Gender and PA Levels in PE

Various studies have shown that males and students who undertake some out-of-school sport activities may be more autonomous and less amotivated to undertake extracurricular PA than females and students who are less physically active. For instance, males were found to report higher levels of autonomous motivation and less amotivation than males within PE context [9,10,11,12,13]. Likewise, Ntoumanis [12] found that physically active students score higher in autonomous motivation and lower in amotivation than non-active students (but see also Viira and Koka and Shen [14,15]). Regarding intentions to undertake extracurricular PA, no clear pattern was found as some researchers found no gender differences [4,12], whereas some others [11] found males to score higher than females. In sum, gender and doing out-of-school sports seem to relate to autonomous motivation and intentions to undertake extracurricular PA. 

We aimed to extend this line of research by examining whether such differences are invariant across different PE motivational environments; that is, whether PE classroom environments attenuate or aggravate students’ differences in autonomous motivation, amotivation, and PA intentions among males versus females and among students who are physical active versus those who are not. We were driven to examine this issue from some prior studies which have shown that perhaps males and females are not equally affected by the social contexts. In his seminal work, for instance, Deci [16] found praise to result in higher intrinsic motivation (as reflected through free-choice behavior) among males but not among females. A similar indirect hint that the context may differentially associate with people’s functioning comes from a few SDT-based diary studies which have shown that some people may benefit more under certain conditions. For example, Moller, Deci, and Elliot [17] revealed that the positive association between day-to-day positive affect and relatedness need satisfaction was even stronger among people who in general had satisfying relationships in their lives. These findings imply that the expected positive associations between determinants of autonomous motivation and optimal functioning may vary, depending on certain situational or personal characteristics. Therefore, more research is needed to uncover under what circumstances who benefits more. 

### 1.3. The Present Research

To pursue our aims, we set up two sets of multilevel models. The first set contained types of motivation (i.e., autonomous motivation, controlled motivation, and amotivation) as dependent variables. In those models, we investigated the degree to which basic need satisfaction, gender and doing out-of-school sport activities predict quality of motivation. Building on this first set of models, the second part examined whether types of motivation (i.e., the dependent variables in the first set of models) predict, along with gender and doing-out-of-school sport activities intentions to undertake extracurricular PA. Moreover, in both sets of models we examined whether the PE classroom climate would predict quality of motivations (i.e., the first set of models) and PA intentions (i.e., the second model). More important, we examined whether PE classroom climate would moderate the relations of need satisfaction, gender, and doing out-of-school sport activities to quality of motivation (first set of models) or the relations between quality of motivation, gender, and doing-out-of-school sport activities to intentions to undertake extracurricular PA (second set of models). 

We formulated the following hypotheses. First, we relied on prior empirical evidence and hypothesized that doing out-of-school sport activities, being male, and experiencing need satisfaction would predict positively autonomous motivation and negatively controlled motivation and amotivation (Hypothesis 1). Similarly, we hypothesized that the same predictors (i.e., sport participation status, gender, and needs satisfaction) along with autonomous motivation would predict positively and that controlled motivation and amotivation predict negatively intentions to undertake extracurricular PA (Hypothesis 2). At the contextual level (i.e., classroom), we hypothesized that need-supportive environment (as defined by the aggregate scores of students’ perceptions of need support) would explain between-class differences in autonomous motivation, controlled motivation, amotivation, and intentions. Specifically, we expected that students who belonged to high need-supportive PE classes would report more autonomous motivation, and intentions and less controlled motivation and amotivation (Hypothesis 3). Finally, as we had no particular hypothesis regarding who benefits more in a need-supportive learning environment, we tested in a more explorative fashion whether need-supportive learning environment would moderate (a) the relations of gender, out-of-school sport activities status, and need satisfaction to autonomous motivation, controlled motivation, and amotivation and (b) the relations of gender, out-of-school sport participation status and the three types of quality of motivation to intentions to undertake extracurricular PA in the future. 

## 2. Materials and Methods 

### 2.1. Participants

Participants of this project were 1346 students nested into 32 randomly selected public compulsory schools situated in the region of Extremadura (Spain). It is a representative sample for the population of Extremadura (n_population_ = 46,231; 95% confidence interval; margin of error = 3%). We excluded data from classes with less than 5 students (to prevent the confounding of level-2 effects) and students who did not fill out all study variables or showed unusual response patterns and response processes (n = 87). The sample of this particular study was composed of 1259 students (44.2% males) aged between 12 to 16 years (*M* = 13.46; *SD* = 0.74) belonging to 100 PE classes (mean number of students per class was 12.59). The average number of PE classes per school was 3.66. 

### 2.2. Instruments

#### 2.2.1. Perceived Need Support

The Questionnaire of Basic Psychological Needs Support in Physical Education [18] was used to asses perceived autonomy, competence, and relatedness support. After reading the statement “In Physical Education classes, my teacher…” the participants rated 12 items, four of which tapping into perceived autonomy support (α = 0.77; e.g., “…often asks us about our preferences with respect to the activities we carry out”), another four competence support (α = 0.71; e.g., “…offers us activities based on our skill level”), and another four relatedness support (α = 0.80; e.g., “…promotes good relationships between classmates at all times”). An overall score of perceived need support was computed by averaging the 12 items (α = 0.88). 

#### 2.2.2. Need Satisfaction

Autonomy, competence, and relatedness need satisfaction were assessed using the Spanish adaptation for PE context [19] of the Basic Psychological Needs in Exercise Scale (BPNES) [20]. This instrument has 12 items (4 items per factor) that follow the initial statement “In my PE class…” and measure the satisfaction of the basic psychological needs of autonomy (α = 0.79; e.g., “…we carry out exercises that are of interest to me”), competence (α = 0.80; e.g., “…I carry out the exercises effectively), and relatedness (α = 0.83; e.g., “…my relationship with my classmates is friendly”). An overall score of need satisfaction was computed by averaging the 12 items (α = 0.88).

#### 2.2.3. Type of Motivation

The different types of behavioral regulations were assessed using the Questionnaire of Motivation in Physical Education Classes (CMEF) [21]. We used 20 items of the questionnaire (4 items per behavioral regulation) that followed the statement “I take part in this PE class…”, exploring: intrinsic motivation (α = 0.83; e.g., “Because PE is fun”), identified regulation (α = 0.81; e.g., “Because I can learn skills that could be used in other areas of my life), introjected regulation (α = 0.76; e.g., “Because I feel bad if I am not involved in the activities), external regulation (α = 0.80; e.g., “Because I want the teacher to think that I am a good student”), and amotivation (α = 0.66; e.g., “But I think that I’m wasting my time with this subject”). 

A confirmatory factor analysis with a five latent factor model yielded acceptable fit (χ2 (160; *N* = 1259) = 504.46, *p* < 0.01, *CFI* = 0.952, *SRMR* = 0.040, *RMSEA* = 0.041 (90% *CI*: 0.037–0.045)). The five factors however failed to accurately reproduce, as expected, the simplex pattern as external regulation was positively related to both intrinsic motivation (*r* = 0.36, *p* < 0.01) and identified regulation (*r* = 0.30, *p* < 0.01) and as amotivation was negatively associated with all the other four types of behavioral regulations, including external regulation (*r* = −0.11, *p* < 0.01). Additionally, after computing a score of autonomous motivation by averaging intrinsic motivation and identified regulation (α = 0.89) and of controlled motivation by averaging introjected and external regulation (α = 0.85), we found in our preliminary analyses a positive, and rather moderate, relation of controlled motivation to perceived need support (*r* = 0.40, *p* < 0.01), need satisfaction (*r* = 0.48, *p* < 0.01), and autonomous motivation (*r* = 0.52, *p* < 0.01). This finding suggested that controlled motivation as assessed through the respective items may not fully capture the theorized construct. Retrospective inspection of the controlled motivation items confirmed our suspicion as most of them tapped into introjected regulation, but not external regulation. We therefore decided to drop controlled motivation from further analyses. 

#### 2.2.4. Extracurricular PA Intentions

To measure students’ intentions to undertake PA outside of the school curriculum, one item was included: “In the coming years, I intend to participate in extracurricular sport/physical activity”. The questionnaire specified that “sport participation” refferred to participating in PA or a sport on a regular basis (at least twice a week). Previous research has implemented single-item scales effectively [3,15,22].

For all of the above questionnaires, participants expressed their level of agreement using a 5-point Likert-type scale that ranged from 1 (strongly disagree) to 5 (strongly agree).

#### 2.2.5. Out-of-school Sport Participation Status

Doing sports as an activity outside of school was assessed through the question: “Do you practice any sport or physical activity outside of school?”. Students had to answer yes or no. The questionnaire clarified that students should answer “yes” if they practiced some form of regular Sport/PA at least twice a week.

### 2.3. Procedure

The present study was approved by the ethical committee of the host university and was supported by the Spanish Professional Association of PE teachers, which enabled us to approach the participating PE teachers. Then, the head researcher contacted the schools to explain the objectives of the study and to request their participation. PE teachers were informed that the purpose of the study and parental consent was also obtained for all participants before commencing the study. All participants were treated according to the ethical guidelines of the American Psychological Association with regards to consent, confidentiality, and anonymity of responses. The participants assented and completed the questionnaire online in the classroom during a class hour via Google Doc, which participants could access via a link provided by the researchers. In all cases, the classrooms were equipped with computers with an internet connection, and each student had approximately 2530– minutes to complete the questionnaires.

### 2.4. Plan of Data Analysis

Initially, descriptive statistics (means and standard deviation) and bivariate correlations between observed variables were computed. Given the nested structure of our data (as students were nested into classrooms, nested into PE teachers), we set up in two steps three separate three-level models. In the first step we examined the degree to which students’ autonomous motivation (Model 1) and amotivation (Model 2) towards PE class-related activities could be explained (a) at the individual level, by students’ need satisfaction as well as by gender, out-of-school sport participation status, and (b) at the classroom level by the aggregate scores of perceived need support. Through the same model we also explored whether perceived need support as some classroom characteristics would moderate the hypothesized associations of gender, out-of-school sport participation status, and need satisfaction to autonomous motivation and amotivation. In the second step, we examined to what extent intentions (Model 3) could be explained, following the same process than models 1 and 2, but including autonomous motivation and amotivation at the individual level (and excluding students’ need satisfaction). All the analyses were conducted through HLM6 software package (version 6, Lincolnwood, Illinois, EE. UU) [23]. 

At the classroom level the aggregate score of perceived need support was entered grand-centered, whereas at the student level gender (0 = males; 1 = females) and out-of-school sport participation status (0 = no; 1 = yes) were entered uncentered, while need satisfaction were entered group-mean centered. Additionally, all the slopes were initially estimated as randomly varying from classroom to classroom and were fixed, in a stepwise fashion, unless they were statistically nonsignificant. No predictors were included at the PE teacher level because we had no particular information about the PE teachers, because the relatively small number of the units at that level (i.e., the PE teachers) might have jeopardized the stability of the coefficients [24], and because we opted for more parsimonious models. 

## 3. Results

### 3.1. Preliminary Analyses

Descriptive statistics and zero-order correlations are presented in Table 1. As can be noticed, females tended to report less out-of-school sport activities and less intentions to undertake such extracurricular PA in the future; also, females reported less need satisfaction and autonomous motivation. Perceived need support, need satisfaction, autonomous motivation as well as intentions to undertake extracurricular PA in the future were all positively intercorrelated and they were all negatively related to amotivation. 

### 3.2. Main Analyses

#### 3.2.1. Autonomous Motivation

Regarding the autonomous motivation model (Model 1) the results are shown in Table 2 (left column). At the student level, need satisfaction related positively to autonomous motivation. This finding provided some support of Hypothesis 1. In addition, gender was negatively (γ_100_ = −0.12, *SE* = 0.04, *p* < 0.01) and out-of-school sport participation status was positively associated (γ_200_ = 0.12, *SE* = 0.05, *p* < 0.05) to autonomous motivation. These findings, addressing reveal that, on average, females and students who were not doing out-of-school sport activities reported lower levels of autonomous motivation. At the classroom level, perceived need support predicted positively, though marginally, autonomous motivation (γ_010_ = 0.53, *SE* = 0.27, *p* = 0.05) providing thus some support to Hypothesis 3. 

As said, classroom’s perceived need support was found to moderate also the relation of gender to autonomous motivation (γ_110_ = 0.67, SE = 0.17, *p* < 0.01). A test of simple slopes indicated that gender was nonsignificant predictor of autonomous motivation in classrooms which were low (i.e., -1 SD below the mean) in perceived need support (γ_100 (-1 SD below the mean in perceived need support)_ = −0.08, SE = 0.08, z = −1.06, *p* > 0.05); instead, it was significant in classrooms which were at average (γ_100 (average perceived need support)_ = −0.12, SE = 0.04, z = −2.81, *p* < 0.01) or high levels (i.e., +1 SD above the mean) in perceived need support (γ_100 (+1 SD above the mean in perceived need support)_ = −0.16, SE = 0.07, z = −2.41, *p* < 0.01). This finding suggests that, other things being equal, males benefit more from need-supportive PE classes than females when the classroom was to some extent need-supportive. Instead, there were no differences between males and females when the classroom was not need-supportive. 

#### 3.2.2. Amotivation

The results for amotivation (Model 2; see Table 2, right panel), indicated, in support of Hypothesis 1, that need satisfaction were negatively related to amotivation (γ_300_ = −0.42, *SE* = 0.05, *p* < 0.01). Unlike the model concerning autonomous motivation, however, gender and out-of-school sport participation status did not predict amotivation. The same was true for the classroom-level predictor, perceived need support, as it failed to predict student-level amotivation (γ_010_ = −0.11, *SE* = 0.19, *p* > 0.05). Therefore, unlike the model concerning autonomous motivation, class-level perceived need support as predictor of amotivation did not provide support in Hypothesis 3.

Yet, classroom-level perceived need support was found to moderate, albeit marginally, the relation between gender and amotivation (γ_110_ = 0.55, *SE* = 0.29, *p* = 0.06) and the relation between need satisfaction and amotivation (γ_310_ = 0.44, *SE* = 0.17, *p* < 0.05). A test of simple slopes for gender showed that females reported less amotivation than males in classes which were high in perceived need support (γ_100 (+1 SD above the mean in perceived need support)_ = −0.26, *SE* = 0.10, z = −2.75, *p* < 0.01). Instead, there were no differences between males and females in amotivation in PE classes which were moderate (γ_100 (average in perceived need support)_ = −0.08, *SE* = 0.06, z = −1.27, *p* > 0.05) or low in perceived need support (γ_100 [-1 SD below the mean in perceived need support]_ = 0.11, *SE* = 0.14, z = 0.82, *p* > 0.05). So, although the previous interaction between gender and need-supportive classes showed that males benefit more than females in terms of autonomous motivation, it seems that need-supportive classes are more beneficial for females in terms that they help them decrease their amotivation. 

Concerning the moderating effect of perceived need support on the relation between need satisfaction and amotivation, a test of simple slopes indicated that the negative relation between need satisfaction and amotivation was stronger among students belonging to classroom which were low in perceived need support (γ_300 (-1 SD velow the mean in perceived need support)_ = −0.57, *SE* = 0.06, z = −9.27, *p* < 0.01) than among students belonging to classroom which were at average (γ_300_ (_average in perceived need support_) = −0.42, *SE* = 0.05, z = −9.10, *p* < 0.01) or high levels of perceived need support (γ_300_ and _(+1 SD above the mean in perceived need support)_ = −0.27, *SE* = 0.09, z = −3.17, *p* < 0.01).

#### 3.2.3. Intentions to Undertake Extracurricular PA in the Future

Moving to the intentions model (Model 3), the results for this model are displayed in Table 3. At the student level, intentions to undertake extracurricular PA in the future were, in support of Hypothesis 2, associated positively with autonomous motivation (γ_300_ = 0.58, *SE* = 0.05, *p* < 0.01) and negatively with amotivation (γ_400_ = −0.10, *SE* = 0.04, *p* < 0.01), after controlling for gender and extracurricular practice; the latter was, positively associated with intentions (γ_200_ = 0.60, *SE* = 0.09, *p* < 0.01). There were also some gender differences in intentions (γ_100_ = −0.16, *SE* = 0.07, *p* < 0.05) suggesting that females, as compared to males, had less intentions to undertake extracurricular PA in the future. 

**Figure 1:**
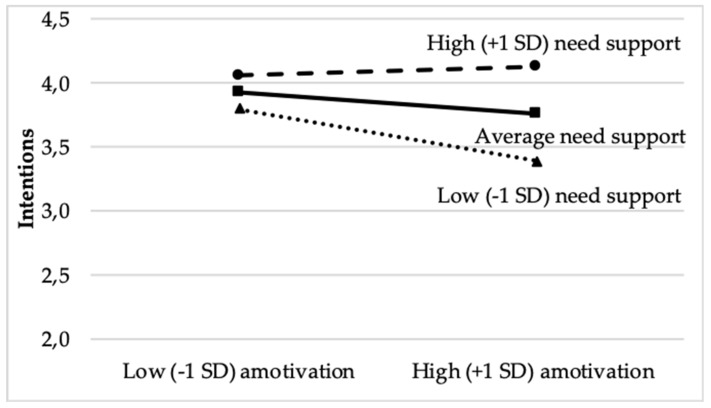
The relation between amotivation and intentions to undertake extracurricular PA as a function of class-level perceived need support.

At the classroom level, perceived need-supportive climate failed to predict intentions (γ_010_ = 0.73, *SE* = 0.44, *p* > 0.05) and thus to provide support to Hypothesis 3. Yet, we found perceived classroom-level perceived need support to moderate the relation between amotivation and intentions to undertake extracurricular PA (γ_010_ = 0.41, *SE* = 0.20, *p* < 0.05). A test of simple slopes showed that the relation between amotivation was significant, and negative, among students belonging to low (i.e., 1 *SD* below the mean) or average (γ_400 (−1 SD below the mean in perceived need support)_ = −0.24, *SE* = 0.07, z = −3.21, *p* < 0.01; and γ_400 (average in perceived need support)_ = −0.10, *SE* = 0.04, z = −2.85, *p* < 0.01, respectively) but not high (1 *SD* above the mean) need-supportive classes (γ_400 (+1 *SD* above the mean in perceived need support)_ = −0.03, *SE* = 0.08, z = 0.49, *p* > 0.05). This finding suggests that high levels of class-level perceived need support attenuated the negative associations between amotivation and intentions to undertake extracurricular PA. A graphical representation of this cross-level interaction is shown in Figure 1.

## 4. Discussion

Grounded in SDT, our primary purpose of this study was to test the relation of the basic psychological needs to motivation (i.e., autonomous motivation and amotivation) and intentions to undertake extracurricular PA activities in the future. Through multilevel analysis we examined, next to need satisfaction as student-level predictor, the role of perceived need support as a classroom-level predictor of autonomous motivation and amotivation. Moreover, we tested the role of gender and out-of-school sport participation status as potential predictors of autonomous motivation, amotivation, and intentions. 

### 4.1. The role of Need Satisfaction and Motivation

In this study we found that students who highly satisfied their needs for autonomy, competence, and relatedness are more likely to engage in PE class-related activities for self-determined motives, and that they are also less likely to feel amotivated. These findings support our hypothesis 1, and are consistent with SDT postulates [5] and previous studies [6], which emphasize the important role of need satisfaction in PE context. Therefore, PE teachers should allocate their resources on developing such teaching strategies that will satisfy students’ autonomy, competence, and relatedness needs. 

Regarding the relation between autonomous motivation and amotivation and future intentions to undertake extracurricular PA, our study showed, as it was hypothesized, autonomous motivation to positively predict and amotivation to negatively predict students’ intentions to undertake extracurricular PA in the future. These findings are consistent with previous researches [3,4,11,12], and they are also in line with a meta-analysis based on the trans-contextual model [25] that also found a significantly association between autonomous motivation in educational contexts on intentions to practice sport and physical activity (β = 0.19, *p* < 0.001). Overall, these results highlight the importance of PE context (particularly motivational regulation) in promoting a physically active lifestyle. 

### 4.2. The Importance of Class’ Perceived Need Support 

At the classroom level, perceived need support positively predicted, albeit marginally, autonomous motivation, whereas it failed to predict amotivation and intentions to undertake extracurricular PA in the future. These findings only confirm Hypothesis 3 with respect to autonomous motivation, and indicate that irrespective of the level of need satisfaction of each student, students who belonged to classrooms that were rated as being need-supportive tended to report that they were autonomous motivated. The importance of a need-supportive environment is also well-established by previous studies. For example, Taylor and Lonsdale [26] found that autonomy support at the classroom level was a positive predictor of competence satisfaction, vitality, and effort. Mouratidis, Vansteenkiste, Sideridis, and Lens [27] also reported that when students experienced a need-supportive teaching style scored higher in interest–enjoyment and vitality than when they experienced a typical (i.e., less need-supportive) teaching style. Regarding lack of relation between class-level need-supportive PE class environment and amotivation, we cannot but speculate that this might be due to the relatively low internal consistency of the scale assessing students’ amotivation and (or) to the relatively low mean scores (i.e., floor effects) in students’ reports of amotivation (41.5% of the students were assigned the lowest possible score—totally disagree—in amotivation). 

With respect to the cross-level interaction between class-level perceived need support on the gender-motivation relations, the results complement prior finding as they show that need-supportive classes were especially beneficial for males in terms of increased autonomous motivation and females in terms of decreased amotivation. These interactions denote that perhaps there might be different routes through which males and females may benefit from a need-supportive environment. However, given the absence of similar finding in the literature more research is needed for this issue. This seems even more imperative because in our research we found no gender differences (i.e., no moderating effects) in the relation between classroom-level perceived need support and intentions. 

With respect to out-of-school sport participation status, we did not find classroom-level perceived need support to moderate the relation of out-of-school sport participation status to any of the dependent variable. This finding suggests that irrespective of whether students participated in out-of-school sport activities or not, they equally benefited from a need-supportive learning environment.

Regarding the relation between need satisfaction and autonomous motivation, our results indicate that this positive association tended to be weaker (although marginal) among students belonging in need-supportive PE classes. In terms of the relation between need satisfaction and amotivation, this was significantly less negative among students belonging to need-supportive PE classes. Furthermore, perceived need support emerged as a moderator of the relation between amotivation and future intentions to undertake extracurricular PA. In PE classes which were high in perceived need support, amotivation was not related negatively to intentions; conversely, in PE classes which were low in need support, the relation between amotivation and intentions was even more negative. Taken together, these findings imply that a need-supportive PE class may buffer somehow the negative relations that one should expect between amotivation and need satisfaction or intentions. Perhaps this is because students who belong to perceived need support PE classes are less likely to become, or remain, amotivated—at least in the short term—even when they fail to fully satisfy their needs. Likewise, such students may still express intentions to undertake extracurricular PA in the future, irrespective of their levels of amotivation. It should be noted however that as we found no classroom effects of perceived need-supportive PE class environment on amotivation or intentions, our explanation here awaits further testing. In any case, our findings imply the role that class environment may play in students’ quality of motivation. To our knowledge, as no other research has tested the moderating effect of perceived need support as a classroom characteristic on the relation of need satisfaction to different types of motivation, more research is needed to examine this issue. 

### 4.3. Gender Differences in Out-of-School Sport Participation 

We also examined the role of gender and out-of-school sport participation status on autonomous motivation and amotivation as well on intentions to undertake extracurricular PA in the future. Regarding gender, results showed, similar to previous studies [9,11,12,13], that boys reported higher scores on autonomous motivation (but not amotivation) and intentions than girls. In terms of autonomous motivation, the results are consistent with previous studies [9,10,11,12,13]. Based on these findings, PE teachers should especially focus on promoting a self-determined motivation on girls, mainly through satisfying their basic psychological needs. For instance, PE teachers may ask them which activities they prefer to exercise during PE class hours, or they may propose activities that are especially tailored to them with respect to ability-difficulty balance. Equally important, they need to facilitate their social relationships. 

In terms of amotivation and the nonsignificant differences that we found, we speculate that although boys may have a higher self-determined motivation than girls, girls are not more amotivated than boys. Although we were not able to include controlled motivation in our study, we speculate that girls may not as much amotivated (i.e., are not bored, or show disinterest and dissatisfaction), but rather they may probably get involved in PE classes for controlled reasons (e.g., to avoid bad feelings, or because this is what they are supposed to do). If so, then PE teachers need to help females’ students internalize their locus of causality and thus further internalize their self-determined motives. In terms of extracurricular PA intentions, our findings were consistent with most previous studies [4,11,12], with boys being more likely to report intentions to undertake extracurricular PA in future. This finding is aligned also with the World Health Organization [28], which has documented that PA levels of males are higher levels than females, suggesting that boys have more intentions to undertake extracurricular PA than females in the following years.

Moreover, the results show that students who participated in out-of-school sport activities reported higher scores in autonomous motivation and intentions to undertake extracurricular PA in the future, although no differences were found in amotivation. Further researches are necessary to clarify this issue, because some prior studies [12] revealed that physically active students scored higher in autonomous motivation and intentions, and lower in amotivation, while other studies [15] found that participation in organized out-of-school sports was related to intentions but not to self-determined motivation, and other studies [14] found no differences between out-of-school sport participants and non-participants in either intentions or self-determined motivation. Our study however, indicated that autonomous motivation (but not amotivation) related (positively) to intentions to undertake extracurricular PA. It seems logical to think that students who practice sport after school time (they enjoy practicing sport) are involved in PE activities by self-determined motives, like enjoyment, and therefore, they have more extracurricular PA intentions in the following years.

### 4.4. Practical Implications

This study highlights the important role of supporting autonomy, competence, and relatedness satisfaction within PE classes. Examples of autonomy support strategies include teachers’ taking perspective of their students’ feelings, encouraging their active participation initiative taking, providing them choices and options, and transferring some freedom and responsibility to them when they perform in-class tasks [7]. With the aim of promoting competence satisfaction, PE teachers can adapt their teaching by offering tasks that are adjusted to the students’ actual level of skills (balancing difficulty-capacity). Further, their feedback must focus on progress by providing task-focused (*r*ather than normative-based) feedback and by acknowledging their students’ effort and improvement. Also, PE teachers should provide sufficient time to all students to they achieve the set objectives [7]. In order to promote relatedness satisfaction, PE teacher are recommended to use a warm and positive communication style; then need to encourage collaborative working, support and respect students’ individuality, and behave in a friendly way. Additionally, PE teacher can use an all-inclusive strategy when groups are formed and promote role-playing or trust activities to improve all students’ feeling of belongingness.

### 4.5. Limitations and Additional Future Directions

A limitation of this study is that controlled motivation was not included in the main analyses, while the learning environment was analyzed through students’ perception about whether their teacher promoted autonomy, competence, and relatedness; so, these problems thereby using ratings from external observers [29]. Additionally, intentions were measured through a single item. Future studies could use broader scales in order to future intentions in more depth (i.e., sport modalities, frequency and intensity of activities, etc.). Further, the study used a cross-sectional methodology and it is observational in nature, so no causal relations can be claimed. It would be interesting if future studies employ a longitudinal design coupled with a quasi-experimental one would test the same hypotheses across time in an intervention versus in a control (i.e., no-treatment) group. Also, some potential cofounders like ethnicity, previous knowledge about PE or parents PA levels were not included, and they could affect the results. Additionally, these findings were found with this particular sample of Spanish students; so, conclusions are not applicable to all students. Lastly, future researchers may consider adding variables of the third level (teacher motivation, teacher need support, teacher need satisfaction, teacher burnout). In our case, this was not recommended due to the low group size of the highest level (n = 32).

## 5. Conclusions

As our study indicates, need satisfaction plays a key role in students’ motivation. It seems that irrespective of gender or investing time and effort in sports, students who fulfill their needs for autonomy, competence, and relatedness in the PE class become more autonomous motivated (especially among males) and less amotivated (especially among females). PE teachers should not only support students’ needs but also pay special attention to students who do not systematically participate in out-of-school sport activities and to females. For these groups, PE teachers need to maximize their students’ basic psychological need satisfaction and eventually their autonomous motivation. 

This study adds to the literature dealing with the interplay between PE learning environments and students’ motivational processes and outcomes. It adds by showing that need supportive learning environments may perhaps not have uniform effects on students’ quality of motivation but may vary depending, among other factors, on students’ gender and need satisfaction. Given that a similar differential effect of need supportive environment was found on intentions to undertake extracurricular PA as a function of students’ amotivation, the current study pinpoints the need for more research that will try to address the question of environmental fit: “Who benefits more (and under what circumstances)”.

## Figures and Tables

**Table 1:** Means, standard deviations, and bivariate correlations of the observed variables.

Variables	1	2	3	4	5	6	7
1. Gender (males vs. females)	-						
2. Out-of-school sport participation	–0.27 **	-					
3. Perceived need support	–0.01	0.05	-				
4. Need satisfaction	–0.10 **	0.20 **	0.67 **	-			
5. Autonomous motivation	–0.17 **	0.22 **	0.59 **	0.70 **	-		
6. Amotivation	0.00	–0.12 **	–0.31 **	–0.39 **	–0.48 **	-	
7. Intentions to undertake extracurricular PA	–0.18 **	0.36 **	0.33 **	0.53 **	0.51 **	–0.31 **	-
*M*	0.56	0.69	4.23	4.03	4.22	1.71	4.17
*SD*	0.50	0.46	0.66	0.68	0.76	0.85	1.14

Note. ** p < 0.01. Gender (0 = males; 1 = females) and out-of-school sport participation status (0 = no; 1 = yes) are dummy coded. PA: physical activity

**Table 2:** The multilevel model predicting autonomous motivation and amotivation.

Fixed Effects		Autonomous Motivation		Amotivation	
		Coefficient	(*SE*)	Coefficient	(SE)
Intercept,	γ_000_	4.25	(0.05)	1.81	(0.07)
*Student-level predictors*					
Gender (males vs. females)	γ_100_	−0.12 **	(0.04)	−0.08	(0.06)
Out-of-school sport participation status,	γ_200_	0.12 *	(0.05)	−0.07	(0.06)
Need satisfaction	γ_300_	0.70 **	(0.03)	−0.42 **	(0.05)
*Classroom-level predictors*					
Need supportive classroom	γ_010_	0.53^05^	(0.27)	−0.11	(0.19)
*Student-level X class-level interactions*					
Need supportive classroom X Gender	γ_110_	0.67 **	(0.17)	0.55^06^	(0.29)
Need supportive classroom X Out-of-school sport participation status	γ_210_	0.04	(0.25)	0.14	(0.17)
Need supportive classroom X Need satisfaction	γ_310_	−0.24^07^	(0.13)	0.44 *	(0.17)
Random effects					
Intercept	r_0*j*_	0.05 **		0.01 **	
Out-of-school sport participation status slope	*r*_2*j*_	0.01 *		-	
Need satisfaction slope	*r*_3*j*_	0.03 **		0.05 **	
Intercept −Need supportive classroom slope	u_00_	-		0.06 **	
Gender − Need supportive slope	u_10_	-		0.04 **	
Student-level variance	ε*_ij_*	0.24		0.53	

*Note*. * *p* < 0.05. ** *p* < 0.01.

**Table 3:** The multilevel model predicting intentions to undertake extracurricular PA.

Fixed Effects		Intentions to Undertake Extracurricular PA	
		Coefficient	(*SE*)
Intercept,	γ_000_	3.84	(0.09)
*Student-level predictors*			
Gender (males vs. females)	γ_100_	–0.16 *	(0.07)
Out-of-school sport participation	γ_200_	0.60 **	(0.09)
Autonomous motivation	γ_300_	0.58 **	(0.05)
Amotivation	γ_400_	–0.10 **	(0.04)
*Classroom-level predictors*			
Need supportive classroom	γ_010_	0.73	(0.44)
*Student-level X class-level interactions*			
Need supportive classroom X Gender	γ_110_	–0.19	(0.44)
Need supportive classroom X out-of-school sport participation status	γ_210_	0.24	(0.43)
Need supportive classroom X Autonomous Motivation	γ_310_	0.23	(0.26)
Need supportive classroom X Amotivation	γ_410_	0.41 *	(0.20)
Random effects			
Intercept	r_0*j*_	0.20 **	
Out-of-school sport participation status slope	*r*_2*j*_	0.24 **	
Autonomous motivation slope	*r*_3*j*_	0.11 **	
Student-level variance	ε*_ij_*	0.77	

*Note*. * *p* < 0.05. ** *p* < 0.05.

## References

[1] Guthold R., A Stevens G., Riley L.M., Bull F.C. (2019). Global trends in insufficient physical activity among adolescents: a pooled analysis of 298 population-based surveys with 1·6 million participants. Lancet Child Adolesc. Heal..

[2] Sallis J.F., McKenzie T.L., Beets M.W., Beighle A., Erwin H., Lee S. (2012). Physical Education’s Role in Public Health: Steps Forward and Backward Over 20 Years and HOPE for the Future. Res. Q. Exerc. Sport.

[3] Sánchez-Oliva D., Sánchez-Miguel P.A., Leo F.M., Kinnafick F.E., García-Calvo T. (2014). Physical Education Lessons and Physical Activity Intentions Within Spanish Secondary Schools: A Self-Determination Perspective. J. Teach. Phys. Educ..

[4] Taylor I.M., Ntoumanis N., Standage M., Spray C.M. (2010). Motivational predictors of physical education students’ effort, exercise intentions, and leisure-time physical activity: a multilevel linear growth analysis. J. Sport Exerc. Psychol..

[5] Ryan R.M., Deci E.L. (2017). Self-Determination Theory: Basic Psychological Needs in Motivation, Development, and Wellness.

[6] Vasconcellos D., Parker P.D., Hilland T., Cinelli R., Owen K.B., Kapsal N., Lee J., Antczak D., Ntoumanis N., Ryan R.M. (2019). Self-determination theory applied to physical education: A systematic review and meta-analysis. J. Educ. Psychol..

[7] Jang H., Reeve J., Deci E.L. (2010). Engaging students in learning activities: It is not autonomy support or structure but autonomy support and structure. J. Educ. Psychol..

[8] Cox A., Duncheon N., McDavid L. (2009). Peers and Teachers as Sources of Relatedness Perceptions, Motivation, and Affective Responses in Physical Education. Res. Q. Exerc. Sport.

[9] De Meyer J., Soenens B., Vansteenkiste M., Aelterman N., Van Petegem S., Haerens L. (2016). Do students with different motives for physical education respond differently to autonomy-supportive and controlling teaching?. Psychol. Sport Exerc..

[10] Haerens L., Aelterman N., Vansteenkiste M., Soenens B., Van Petegem S. (2015). Do perceived autonomy-supportive and controlling teaching relate to physical education students’ motivational experiences through unique pathways? Distinguishing between the bright and dark side of motivation. Psychol. Sport Exerc..

[11] Lim B.C., Wang C.J. (2009). Perceived autonomy support, behavioural regulations in physical education and physical activity intention. Psychol. Sport Exerc..

[12] Ntoumanis N. (2005). A Prospective Study of Participation in Optional School Physical Education Using a Self-Determination Theory Framework. J. Educ. Psychol..

[13] Ferriz R. (2013). Predicting Satisfaction in Physical Education Classes: A Study Based on Self-Determination Theory. Open Educ. J..

[14] Viira R., Koka A. (2012). Participation in afterschool sport: Relationship to perceived need support, need satisfaction, and motivation in physical education. Kinesiology.

[15] Shen B. (2014). Outside-school physical activity participation and motivation in physical education. Br. J. Educ. Psychol..

[16] Deci E.L. (1972). Intrinsic motivation, extrinsic reinforcement, and inequity. J. Pers. Soc. Psychol..

[17] Moller A.C., Deci E.L., Elliot A.J. (2010). Person-Level Relatedness and the Incremental Value of Relating. Pers. Soc. Psychol. Bull..

[18] Sánchez-Oliva D., Leo F.M., Amado D., Cuevas R., García-Calvo T. (2013). Desarrollo y validación del cuestionario de apoyo a las necesidades psicológicas básicas en educación física [Development and validation of the questionnarie of basic psychological need support in physical education]. Mot. Eur. J. Hum. Mov..

[19] Moreno J.A., González-Cutre D., Chillon M., Parra N. (2008). Adaptación a la educación física de la escala de las necesidades psicológicas básicas en el ejercicio [Adaptation of the basic psychological needs in exercise scale to physical education]. Rev. Mex. Psicol..

[20] Vlachopoulos S.P., Michailidou S. (2006). Development and Initial Validation of a Measure of Autonomy, Competence, and Relatedness in Exercise: The Basic Psychological Needs in Exercise Scale. Meas. Phys. Educ. Exerc. Sci..

[21] Sánchez-Oliva D., Amado D., Leo F.M., González-Ponce I., García-Calvo T. (2012). Desarrollo de un cuestionario para valorar la motivación en educación física [Development of a questionnaire to assess the motivation in physical education]. Rev. Iberoam. Psicol. Del. Ejerc y el Deport..

[22] Ntoumanis N. (2001). A self-determination approach to the understanding of motivation in physical education. Br. J. Educ. Psychol..

[23] Raudenbush S.W., Bryk A.S., Congdon R. (2004). HLM 6 for Windows [Computer software].

[24] Maas C.J., Hox J. (2005). Sufficient Sample Sizes for Multilevel Modeling. J. Res. Methods Behav. Soc. Sci..

[25] Hagger M.S., Chatzisarantis N.L.D. (2016). The Trans-Contextual Model of Autonomous Motivation in Education: Conceptual and Empirical Issues and Meta-Analysis. Rev. Educ. Res..

[26] Taylor I.M., Lonsdale C. (2010). Cultural differences in the relationships among autonomy support, psychological need satisfaction, subjective vitality, and effort in British and Chinese physical education. J. Sport Exerc. Psychol..

[27] Mouratidis A.A., Vansteenkiste M., Sideridis G., Lens W. (2011). Vitality and interest–enjoyment as a function of class-to-class variation in need-supportive teaching and pupils’ autonomous motivation. J. Educ. Psychol..

[28] World Health Organization WHO Global InfoBase. https://www.who.int/data/gho.

[29] Haerens L., Aelterman N., Berghe L.V.D., De Meyer J., Soenens B., Vansteenkiste M. (2013). Observing physical education teachers’ need-supportive interactions in classroom settings. J. Sport Exerc. Psychol..

